# Burden of Respiratory Syncytial Virus Infection in South African Human Immunodeficiency Virus (HIV)-Infected and HIV-Uninfected Pregnant and Postpartum Women: A Longitudinal Cohort Study

**DOI:** 10.1093/cid/cix1088

**Published:** 2017-12-15

**Authors:** Shabir A Madhi, Clare L Cutland, Sarah Downs, Stephanie Jones, Nadia van Niekerk, Eric A F Simoes, Marta C Nunes

**Affiliations:** 1 Respiratory and Meningeal Pathogens Research Unit, Medical Research Council; 2 Department of Science and Technology, National Research Foundation, Vaccine Preventable Diseases, University of the Witwatersrand, Johannesburg, South Africa; 3 Department of Pediatrics, University of Colorado School of Medicine; 4 Center for Global Health, Colorado School of Public Health, Aurora

**Keywords:** RSV, pregnant women, infant, vaccine, epidemiology

## Abstract

**Background:**

Limited data exist on the burden of respiratory syncytial virus (RSV) illness among pregnant women, to determine their potential benefit from RSV vaccination. We evaluated the incidence of RSV illness from midpregnancy until 24 weeks postpartum in human immunodeficiency virus (HIV)–uninfected and HIV-infected women and their infants.

**Methods:**

Mother–infant dyads were enrolled in maternal influenza vaccine efficacy trials. These included 1060 and 1056 HIV-uninfected pregnant women in 2011 and 2012, respectively, 194 HIV-infected pregnant women in 2011, and their infants. Upper respiratory tract samples obtained at illness visits were tested for RSV.

**Results:**

The incidence (per 1000 person-months) of RSV illness (n = 43 overall) among HIV-uninfected women was lower in 2011 (1.2; 95% confidence interval [CI], .6–2.2) than in 2012 (4.0; 95% CI, 2.8–5.6). The incidence of RSV illness (n = 5) in HIV-infected women was 3.4 (95% CI, 1.4–8.1). Maternal RSV infection was associated with respiratory symptoms including cough (72.1%), rhinorrhea (39.5%), sore throat (37.2%), and headache (42%), but fever was absent. RSV infection during pregnancy was not associated with adverse pregnancy outcomes. Postpartum, RSV infection in mothers (n = 27) was associated with concurrent infection among 51.9% of their infants and, conversely, 29.8% of mothers investigated within 7 days of their infants having an RSV illness also tested positive for RSV.

**Conclusions:**

RSV infection is associated with respiratory illness during pregnancy and postpartum. Vaccination of pregnant women against RSV could benefit the mother, albeit primarily against nonfebrile illness, and her infant.

**Clinical Trial Registration:**

NCT01306669 and NCT01306682.

## Teaser

Respiratory syncytial virus (RSV) infection was associated with respiratory illness during pregnancy and postpartum in women. Vaccination during pregnancy against RSV could benefit the mother, primarily against nonfebrile illness, and her infant.

**(See the Editorial Commentary by Polack on pages 1666–7.)**

Respiratory syncytial virus (RSV) is a leading cause of under-5 morbidity and mortality, causing an estimated 48000–74500 under-5 childhood deaths in 2015, 50% occurring during the first 6 months of life [1]. An inverse association has been reported between maternal RSV neutralizing antibodies and risk for RSV hospitalization among young infants [2, 3]. Furthermore, passive immunization of premature infants with a monoclonal antibody (palivizumab) protects infants against RSV lower respiratory tract infections (LRTIs) [4]. This has served as a rationale for development of RSV vaccine candidates targeted at pregnant women to enhance transplacental antibody transfer to their infants [5], including one currently in phase 3 trials (ClinicalTrials.gov identifier NCT02624947). Influenza vaccination during pregnancy has been shown to prevent influenza illness among pregnant women and protect their young infants [6, 7]. Similarly, maternal pertussis vaccination prevents pertussis in the infants of vaccinated women [8].

The ethical acceptability of vaccination of pregnant women primarily aimed at protecting their infants can be mitigated should there be a direct benefit to the women themselves. Consequently, establishing the burden of RSV illness during pregnancy and the postpartum period in women, and investigating whether RSV infection in pregnant women affects pregnancy outcomes, could strengthen the case for vaccination of pregnant women. Recent studies reported an overall incidence of RSV in pregnant women of 3.9 per 1000 person-years in Nepal, and 0.03 per 1000 person-days in Mongolia, with only limited numbers of RSV-infected women having been identified (14/3693 [0.4%] in Nepal and 4/1260 [0.3%] in Mongolia) [9, 10]. Furthermore, there was a recent report on 3 pregnant women with RSV in the United States, 2 requiring mechanical ventilation [11]. The incidence of severe disease associated with RSV infection, however, remains undefined.

The aim of this study was to evaluate the burden of confirmed RSV illness from midpregnancy to 24 weeks postpartum among cohorts of human immunodeficiency virus (HIV)–uninfected and HIV-infected black-African women to determine their potential benefit from maternal RSV vaccination aimed primarily at protecting their probands.

## METHODS

This study reports on retrospective testing of samples that were prospectively collected in 2 cohorts of HIV-uninfected pregnant women enrolled from March to August 2011 (n = 1056) and March to July 2012 (n = 1060), as well as 194 HIV-infected pregnant women enrolled from March to June in 2011. The mother–infant dyads were enrolled into double-blind randomized controlled trials (1:1 randomization to trivalent inactivated influenza vaccine [IIV] or saline placebo) that evaluated the safety and efficacy of IIV during pregnancy against influenza illness in the women and their infants as described previously [6]. The women were enrolled from midpregnancy and followed up until 24 weeks postpartum. Furthermore, live births were followed up until 24 weeks of age.

### Sample Collection and Testing

Details on the methods used for surveillance and sample collection have been published previously [6]. In brief, women from the time of enrollment, and infants from time of birth, were followed up weekly by either in-person visits or telephone calls to elicit the presence of respiratory symptoms. Those fulfilling predefined criteria for influenza-like illness (ILI) were requested to attend the clinic for respiratory sampling (Supplementary Materials). Furthermore, all participants seeking medical care for any unsolicited respiratory illness, irrespective of duration of symptoms or presence of fever, were also sampled. Respiratory samples were also collected from participants hospitalized for any acute cardiopulmonary illness. Sampling of respiratory secretions included nasopharyngeal aspirates in infants obtained by douching the nasopharynx with 2 mL of 0.9% sterile saline that was then suctioned and collected in a sterile container with universal transport medium (UTM; Copan, Brescia, Italy), and oropharyngeal plus flocked nasopharyngeal swabs in the women that were placed in UTM. Samples were transported to the Respiratory and Meningeal Pathogens Research Unit laboratory at Chris Hani-Baragwanath Academic Hospital, University of the Witwatersrand where testing was undertaken for influenza virus as described previously [6]. The residual sample was stored and subsequently tested for RSV. Two-step real-time polymerase chain reaction (PCR) was performed on the archived specimens using TaqMan technology and the primers and probes specific for RSV-A and RSV-B (LTC.Tech South Africa [Pty], Ltd) that have been previously published [12]. Amplification data was analyzed with Applied Biosystems 7500 software (Foster City, California) with manually defined thresholds. Negative samples were defined as those with cycle threshold (Ct) values >37. If participants had recurrent illness visits, only visits occurring at least 15 days apart were included in the analysis.

### Statistical Analysis

**Figure 1.**
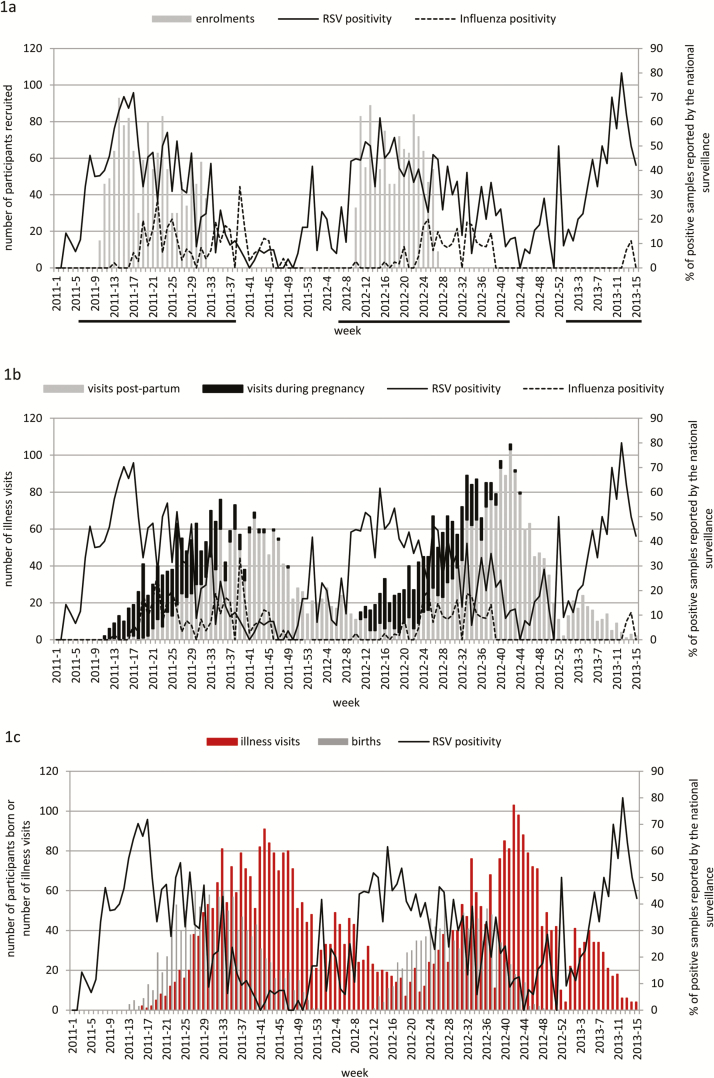
Number of participants in the study by week and respiratory syncytial virus (RSV) and influenza seasonality. RSV and influenza positivity rate were calculated using data from the South Africa national surveillance system. Underlined are the weeks considered part of the RSV season. *A*, Enrollment of pregnant women into the study. *B*, Maternal study illness visits during pregnancy and postpartum. *C*, Infants born from mothers in the study and infant study illness visits.

Due to the established seasonality of influenza virus in the study area, the IIV efficacy trial aimed to enroll pregnant women prior to the expected onset of the influenza season, based on past epidemiological data available from the National Institute for Communicable Diseases (NICD) [13]. This resulted in pregnant women mainly being enrolled during the anticipated RSV season, which generally precedes the onset of the influenza season in Johannesburg (Figure 1A and 1B). Consequently, the majority of births occurred after the peak of the RSV season and were concentrated during the influenza season (Figure 1C). For the purposes of the current analysis, the RSV season was defined as having started the first day of the first of 4 consecutive weeks of that year in which at least 10% of tested samples at NICD were positive for RSV, and having ended at the end of the last week when, after that, <10% of the samples were positive for RSV for 2 consecutive weeks. These data were also used to calculate the person-time (per 1000 person-months) of RSV exposure.

Categorical variables were described as proportions and compared by χ^2^ test or Fisher exact test, and continuous variables were represented as means and compared by Student *t* test. Incidence was calculated as density incidence using person-time as denominator; 95% confidence intervals (95% CIs) for incidence estimates were calculated using a Poisson distribution. Follow-up was censored at last contact (if infant was <175 days old), death (if occurred before infant was <175 days old), when infant was 175 days old, or at the first RSV PCR–positive episode, whichever came first. *P* values <.05 were considered significant. Analyses were performed using Stata version 13.1 software (StataCorp, College Station, Texas).

### Ethical Considerations

The initial trials and subsequent analysis were approved by the Human Research Ethics Committee of the University of the Witwatersrand (reference numbers 101106 and 101107) and conducted in accordance with Good Clinical Practice guidelines. Mothers provided written informed consent for themselves and their infants. The original trials were registered on ClinicalTrials.gov (NCT01306669 and NCT01306682).

## RESULTS

### Characteristics of the Study Population

Detailed demographic information of the study population has been published previously [6]. The 2011 and 2012 cohorts of HIV-uninfected women were similar in demographic characteristics and clinical course of their pregnancy and outcomes of their infants (Table 1). Similarly, in 2011 the HIV-uninfected and HIV-infected women had similar demographic characteristics.

**Table 1.** Demographic Characteristics of the Study Participants According to Human Immunodeficiency Virus Status and Year of Enrollment

Characteristic	HIV-Infected (n = 194)	HIV-Uninfected, 2011 (n = 1060)	*P* Value^a^	HIV-Uninfected, 2012 (n = 1056)	*P* Value^b^
Women					
IIV group, No. (%)	100 (51.6)	532 (50.2)	.73	530 (50.2)	.99
Mean age at enrollment (SD), y	28.2 (5.1)	26.2 (5.3)	<.001	26.1 (5.3)	.65
Mean gestational age at enrollment (SD), wk	27.3 (3.8)	26.9 (4.3)	.21	26.7 (4.4)	.58
Mean body mass index at enrollment (SD), kg/m^2^	28.8 (5.1)	28.9 (5.8)	.83	28.4 (5.8)	.07
Mean overall study follow-up time (SD), mo	7.8 (1.5)	8.0 (1.8)	Not done	8.0 (1.7)	Not done
Mean follow-up time during RSV season (SD), mo	4.9 (0.9)	4.5 (1.3)		4.5 (1.3)	
Mean follow-up time while pregnant during RSV season (SD), mo	2.5 (1.0)	2.4 (1.0)		2.6 (1.2)	
Mean follow-up time postpartum during RSV season (SD), mo	2.5 (1.2)	2.2 (1.2)		4.0 (0.9)	
Deaths, No. (%)	2 (1.0)	2 (0.2)	.056	0	.50
Infants	HIV-Exposed (n = 188)	HIV-Unexposed (n = 1028)	*P* Value^a^	HIV-Unexposed (n = 1021)	*P* Value^b^
Preterm (<37 wk gestational age) birth, No. (%)	26 (13.8)	70 (6.8)	.001	134 (13.1)	<.001
Mean birthweight (range), kg	2.9 (0.8–4.3)	3.1 (0.5–4.6)	.007	3.0 (0.6–4.8)	.18
Birthweight <2500 g, No. (%)	29 (15.6)	118 (11.5)	.12	137 (13.4)	.20
Male sex, No. (%)	96 (51.6)	534 (52.0)	.92	546 (53.5)	.50
Mean overall study follow-up time (SD), mo	5.2 (1.3)	5.4 (0.9)	Not done	5.5 (0.8)	Not done
Mean follow-up time during RSV season (SD), mo	2.3 (1.3)	2.1 (1.2)		3.9 (1.0)	
Deaths, No. (%)	14 (7.5)	18 (1.8)	<.001	18 (1.8)	.98

Abbreviations: HIV, human immunodeficiency virus; IIV, trivalent inactivated influenza vaccine; RSV, respiratory syncytial virus; SD, standard deviation.

^a^Comparing HIV-infected to HIV-uninfected women or HIV-exposed to HIV-unexposed infants from the 2011 cohort.

^b^Comparing HIV-uninfected cohorts from 2011 and 2012.

Using data available from the NICD for Johannesburg, the enrollment of the pregnant women coincided with the RSV epidemics in 2011 and 2012 (Figure 1A). The peak in number of respiratory visits among the women, including the postpartum period, occurred mainly during the influenza season (Figure 1B). Furthermore, 77.5% of births in 2011 and 97.8% in 2012 occurred during the RSV season. Details on the total number of illness visits, as well as visits stratified by the RSV season and availability of samples for RSV testing, are reported in Supplementary Table 1.

### RSV Illness in Pregnant and Postpartum Women

The overall attack rate of RSV-associated illness did not differ between IIV and placebo recipients among the HIV-uninfected women in 2011 (1.5% [8/532] vs 0.4% [2/528], respectively; *P* = .11) and 2012 (2.8% [15/530] vs 3.4% [18/526], respectively; *P* = .58), and similarly so in the HIV-infected women (4.0% [4/100] vs 1.1% [1/94], respectively; *P* = .37). Hence, the IIV and placebo groups were combined in further analyses.

Of the sampled illness visits occurring during the RSV seasons, 93.6% (248/265) of samples among HIV-infected women, and 94.9% (798/841) in HIV-uninfected women from the 2011 cohort, were available for RSV testing (Supplementary Table 1).

There were 5 RSV illnesses among the 2011 cohort of HIV-infected women (2.6% of 194), including 3 RSV-A and 2 RSV-B subtypes. Four of these cases were identified during the defined RSV-epidemic period. In the 2011 HIV-uninfected cohort, 10 of the 1060 women had an RSV illness (0.9%; *P* = .07 compared with HIV-infected women), including 7 RSV-A and 3 RSV-B subtypes; 9 of these visits occurred during the RSV season. The incidence (per 1000 person-months) of RSV illness during the 2011 RSV season was 4.3 in HIV-infected and 1.9 in HIV-uninfected women and 6.6 and 1.7, respectively, during pregnancy (incidence rate ratio [IRR], 4.0 [95% CI, .9–17.8]). The incidence of RSV illness during the postpartum period was identical in HIV-infected and HIV-uninfected women (2.3 per 1000 person-months; Table 2). The mean Ct value in HIV-infected women (29) was lower than in HIV-uninfected women (32), albeit not significant (*P* = .07).

**Table 2.** Incidence of Respiratory Syncytial Virus–Associated Illness in Human Immunodeficiency Virus (HIV)–Infected and HIV-Uninfected Women and Their Infants

Group	Rate	Incidence^a^ (95% CI)	Rate	Incidence^a^ (95% CI)	*P* Value^b^	Rate	Incidence^a^ (95% CI)	*P* Value^c^
	HIV-Infected (n = 194)		HIV-Uninfected, 2011 (n = 1060)			HIV-Uninfected, 2012 (n = 1056)		
Women: during RSV season only								
Overall	4 (2.1%)	4.3 (1.6–11.4)	9 (0.9%)	1.9 (1.0–3.7)	.18	29 (2.8%)	4.3 (3.0–6.2)	.032
During pregnancy	3 (1.6%)	6.6 (2.1–20.4)	4 (0.4%)	1.7 (.6–4.4)	.07	14 (1.3%)	5.3 (3.1–8.9)	.041
Postpartum	1 (0.5%)	2.3 (.3–16.1)	5 (0.5%)	2.3 (1.0–5.5)	.99	15 (1.4%)	3.8 (2.3–6.3)	.33
Women: overall study period								
Overall	5 (2.6%)	3.4 (1.4–8.1)	10 (0.9%)	1.2 (.6–2.2)	.059	33 (3.1%)	4.0 (2.8–5.6)	.001
During pregnancy	3 (1.6%)	6.5 (2.1–20.2)	4 (0.4%)	1.5 (.6–4.0)	.053	14 (1.3%)	5.3 (3.1–8.9)	.026
Postpartum	2 (1.0%)	2.0 (.5–8.0)	6 (0.6%)	1.1 (.5–2.4)	.45	19 (1.8%)	3.4 (2.2–5.3)	.015

Abbreviations: CI, confidence interval; HIV, human immunodeficiency virus; RSV, respiratory syncytial virus.

^a^Incidence was calculated as number of cases per 1000 person-months.

^b^HIV-infected vs HIV-uninfected enrolled in 2011.

^c^HIV-uninfected enrolled in 2011 vs HIV-uninfected enrolled in 2012.

Comparing HIV-uninfected women from 2011 and 2012, a lower percentage of illness samples obtained during RSV season were available for RSV testing from the 2012 cohort (87.5% [1460/1669] vs 94.9% [798/841]; *P* < .001). Nevertheless, a greater percentage of women had RSV-associated illness from the 2012 cohort compared with the 2011 cohort overall (3.1% vs 0.9%; *P* < .001), as well as during the RSV season (2.8% vs 0.9%; *P* = .001) (Table 2). Of the 33 RSV illnesses identified from the 2012 cohort, there were 25 cases of RSV-A and 9 cases of RSV-B, with 1 episode having both subtypes identified simultaneously.

Among all the HIV-uninfected women, 18 of the 43 (41.9%) RSV cases were identified from visits occurring during pregnancy and the remainder occurred in the postpartum period. The incidence of RSV (per 1000 person-months) during the RSV season was higher among the 2012 cohort compared with the 2011 cohort overall (4.3 vs 1.9, respectively; IRR, 2.3 [95% CI, 1.1–4.8]) and during pregnancy (5.3 vs 1.7, respectively; IRR, 3.2 [95% CI, 1.0–9.7]) (Table 2).

Notably, among all the HIV-uninfected women, only 2 of 348 (0.6%) samples obtained during visits which fulfilled the ILI criteria tested positive for RSV, compared to 41 of 2802 (1.5%) medically attended non-ILI illness visits (*P* = .22; Supplementary Table 1). Furthermore, none of the women with RSV infection presented with fever, although 2 presented with chills. The common symptoms associated with RSV infection included cough (72.1%), headache (41.9%), rhinorrhea (39.5%), and sore throat (37.2%). There was no difference in the prevalence of these symptoms between illnesses associated with RSV infection compared with those associated with influenza infection, or illness in which neither of the 2 viruses was identified. The only symptom that differed between RSV and influenza-associated illness was a higher prevalence of myalgia in influenza infection episodes (19.3%) compared with RSV (4.7%) (*P* = .037; Table 3). Two (4.7%) of the RSV infections among the women were associated with pneumonia, similar to the number among women with influenza infection (3/57 [5.5%]). None of the RSV illnesses among the women required hospitalization. The mean Ct value of the 2 HIV-uninfected women with RSV-associated pneumonia was 35 (33 and 37), and 32 in those who did not develop pneumonia (*P* = .35).

**Table 3.** Clinical Signs and Symptoms of Respiratory Syncytial Virus and Influenza Illness in Pregnant and Postpartum Human Immunodeficiency Virus–Uninfected Women

Characteristic	RSV-Infected Women (n = 43)	Influenza-Infected Women (n = 57)	Neither RSV nor Influenza Virus Identified (n = 2019)	*P* Value^a^	*P* Value^b^
Mean age at enrollment (SD), y	26.1 (5.6)	26.7 (5.8)	25.6 (5.3)	.62	.57
Primigravida, No. (%)	10 (23.3)	15 (26.3)	696 (34.5)	.73	.13
Hospitalized for LRTI within 15 d of viral detection, No. (%)	0	1 (1.8)	2 (0.1)^c^	.99	.99
Signs and symptoms, No. (%)^d^	(n = 43)	(n = 57)	(n = 3348)		
Cough	31 (72.1)	47 (82.5)	2234 (66.7)	.22	.50
Chills/rigors	4 (9.3)	13 (22.8)	407 (12.2)	.11	.81
Rhinorrhea	17 (39.5)	19 (33.3)	1180 (35.2)	.52	.64
History of fever	0	4 (7.0)	55 (1.6)	.13	.99
Myalgia	2 (4.7)	11 (19.3)	262 (7.8)	.037	.77
Headache	18 (41.9)	28 (49.1)	1610 (48.1)	.47	.41
Sore throat	16 (37.2)	26 (45.6)	1255 (37.5)	.40	.94
Pneumonia	2 (4.7)	3 (5.5)	137 (4.4)	.99	.72
Bronchitis	0	1 (1.8)	11 (0.4)	.99	.99
Pulmonary tuberculosis	0	1 (1.8)	0	.99	…

Two illness episodes had both RSV and influenza detected simultaneously, and 3 women with influenza infection did not have samples available for further RSV testing.

Abbreviations: LRTI, lower respiratory tract infection; RSV, respiratory syncytial virus; SD, standard deviation.

^a^RSV-associated illnesses vs influenza-associated illnesses.

^b^RSV-associated illnesses vs neither RSV nor influenza-associated illness.

^c^All women who did not have an RSV or influenza episode were included.

^d^Based on the number of samples available for testing.

Investigating for pregnancy outcomes among HIV-uninfected women in whom an RSV illness occurred during pregnancy (n = 18), the prevalence of preterm birth (11.1%) and low birthweight (5.6%) was similar to those in women with an influenza infection during pregnancy (9.4% for each), as well as compared to women who did not have either RSV or influenza infection during pregnancy (9.1% and 10.8%, respectively). Also, no diagnosed maternal RSV infection was temporally related to stillbirth (n = 0; Table 4).

**Table 4.** Pregnancy Outcomes in Human Immunodeficiency Virus–Uninfected Women

Pregnancy Outcome	RSV Illness During Pregnancy (n = 18)	Influenza Illness During Pregnancy (n = 32)	Neither RSV or Influenza Illness During Pregnancy (n = 1980)^a^	*P* Value^b^	*P* Value^c^
Stillbirth	0	0	32 (1.6)	…	.99
Preterm birth	2 (11.1)^d^	3 (9.4)	181/1949 (9.1)	.99	.68
Birthweight <2500 g	1 (5.6)	3 (9.4)	213/1946 (10.8)	.99	.71

Abbreviation: RSV, respiratory syncytial virus.

^a^Women with known pregnancy outcomes who did not have an RSV or influenza-associated illness during pregnancy.

^b^RSV-associated illnesses vs influenza-associated illnesses.

^c^RSV-associated illnesses vs neither RSV nor influenza-associated illnesses.

^d^One woman delivered a 32-week preterm 15 days after having an RSV illness; 1 woman delivered a 36-week preterm 112 days after having an RSV illness.

Details of the clinical presentations of RSV illness among the HIV-infected women are presented in Supplementary Table 2. The few cases of RSV (n = 5) among the HIV-infected women limited further comparisons; notably, none of these cases warranted hospitalization or presented as pneumonia.

### RSV Illness in the Infants

There was no difference in the rates of RSV-associated illness in infants born to IIV and placebo recipients in either the HIV-unexposed cohorts in 2011 (5.9% [30/513] vs 4.9% [25/515], respectively; *P* = .48) or 2012 (6.6% [34/513] vs 4.9% [25/508], respectively; *P* = .24), nor the 2011 HIV-exposed cohort (9.0% [9/100] vs 8.0% [7/88], respectively; *P* = .37). Hence, the IIV and placebo groups were combined in further analyses.

The peak number of illness visits among the infants occurred primarily after the RSV seasons and during the influenza seasons (Figure 1C). Two HIV-unexposed and 2 HIV-exposed infants had 2 RSV-associated illness spaced 26, 29, 21, and 32 days apart, respectively. As these episodes involved the same RSV subtype in the infants and the PCR Ct was consistently higher for the second detection, we considered these to be single RSV episodes. Overall, RSV was identified from 16 (8.5%) HIV-exposed infants and 114 (5.4%) HIV-unexposed infants.

Among HIV-unexposed infants, RSV illnesses were more likely to present as LRTI (17.6%) than illnesses where RSV was not detected (8.0%) (*P* = .001). Also, infants with RSV-associated illness were 4.6-fold (95% CI, 2.4- to 8.6-fold) more likely to be hospitalized within 15 days of the illness (9.6%) than those without RSV illness (2.1%) (*P* < .001; Table 5. The mean Ct value among HIV-unexposed RSV cases that presented with LRTI (26) was similar to that among those who did not develop LRTI (27). Similarly, among infants with RSV illness, the mean Ct values were similar between hospitalized and nonhospitalized cases in HIV-unexposed (25 vs 27; *P* = .21) and HIV-exposed (27 vs 29; *P* = .53) infants.

**Table 5.** Respiratory Syncytial Virus Illness in Human Immunodeficiency Virus (HIV)–Unexposed and HIV-Exposed Infants

Characteristic	RSV Illness (n = 114)	No RSV Illness (n = 1935)	*P* Value
HIV-unexposed infants			
Hospitalized for LRTI within 15 d of viral detection	11 (9.7)	41 (2.1)^a^	<.001
Deaths	0	36 (1.9)	.26
Presented with LRTI^b^	19/108 (17.6)	245/3063 (8.0)	<.001
HIV-exposed infants	**(n = 16)**	**(n = 172)**	
Hospitalized for LRTI within 15 d of viral detection	3 (18.8)	11 (6.4)^a^	.10
Deaths	1 (6.3)^c^	13 (7.6)	.99
Presented with LRTI^b^	2/17 (11.8)	35/324 (10.8)	.90

Data are presented as No. (%) unless otherwise indicated.

Abbreviations: HIV, human immunodeficiency virus; LRTI, lower respiratory tract infection; RSV, respiratory syncytial virus.

^a^All infants who did not have an RSV episode were included.

^b^Based on the number of samples available for testing.

^c^Sixteen-day-old girl visited the study clinic 4 days before death with cough, blocked nose, and noisy breathing; she was found to have coryza, with no signs of sepsis or respiratory distress, sent home on antibiotics, deteriorated at home, and was dead on arrival at hospital.

Fifty-nine mothers of the 130 (45.4%) infants with RSV infection also had a respiratory illness within 1 week of the infant’s illness. Samples for RSV testing were available for 47 (79.7%) of these mothers, among whom RSV was detected in 29.8% (n = 14; 12 HIV-uninfected and 2 HIV-infected) of illnesses. This included 11 episodes in which RSV was detected on the same day in the mother–infant pair and 3 first in the infant (3–7 days before maternal detection).

## DISCUSSION

In our study, RSV infection was 2- to 4-fold more common during the latter half of pregnancy and up until 24 weeks postpartum than previously reported from a similar Nepalese birth cohort [9]. The incidence (per 1000 person-months) of RSV infection in our study among HIV-uninfected women fluctuated year to year, being greater for the 2012 cohort (4.3) than the 2011 cohort (1.9); including during pregnancy (5.3 vs 1.7). Furthermore, although not significant possibly due to the limited sample size, HIV-infected women had a higher incidence of RSV illness (4.3 vs 1.9), including during pregnancy (6.6 vs 1.7).

The most likely reason for the higher incidence of RSV infection observed in our study compared with the study from Nepal during RSV season (0.98 per 1000 person-months), possibly relates to differences in the threshold used for investigating for respiratory illness. Testing in Nepal was limited to women with respiratory illness who reported or measured fever (>38°C) and at least 1 other ILI symptom of cough, myalgia, sore throat, or rhinorrhea [9]. In contrast, in addition to investigating such cases, we also investigated for other unsolicited medically attended respiratory illnesses, irrespective of whether they fulfilled the ILI criteria. The majority of RSV cases identified in our study did not fulfil the ILI criteria, and in fact none of the women volunteered a history of fever or were pyrexial at the time of investigation for that illness. Consequently, the Nepal study, as postulated by the authors, likely underestimated the incidence of RSV infection among the pregnant and postpartum women. In the other published study on RSV during pregnancy from Mongolia, similar to the Nepalese study, only women with fever and a diagnosis of ILI were tested for RSV. This study only documented 4 cases of RSV infection among 1260 (0.3%) pregnant women, with an estimated incidence of 0.03 per 1000 person-days during the sampling period. The detection of RSV in this study was likely compromised by the use of a rapid point-of-care diagnostic test, which has reduced sensitivity compared to PCR used in our study [10].

Although the women with RSV infection in our study did not present with fever, the RSV illness was nevertheless sufficiently severe for them to seek medical attention. Furthermore, these RSV infections were commonly associated with other systemic symptoms including headache and symptoms of upper respiratory tract infections. The low prevalence of fever in RSV illness was documented since the mid-1970s by Hall et al, being present in only 5%–27% of household cases with RSV infection [14]. Further reasons for the difference in incidence observed between these studies could be due to geographic and temporal differences in the intensity and virulence of the circulating viruses between the countries. Even within our study, we observed significant year-to-year variability in incidence of RSV illness, which could have been due to more intense circulation of RSV during 2012 than 2011. Nevertheless, as our surveillance was focused on sampling for ILI episodes, although sampling of unsolicited illness visits was undertaken, these unsolicited medically attended visits of the mother and her infant was done at the discretion of the mother. Consequently, milder episodes of RSV illness for which the mother chose not to seek medical attention could have been missed and consequently underestimated the incidence of RSV in our study.

A case-series of RSV infection in 2 pregnant women from the United States reported on them requiring mechanical ventilation [11], and the need to further investigate the severity of RSV infection among pregnant women. In our study, 2 (4.7%) of the RSV infections among HIV-uninfected women were associated with pneumonia, from which both women recovered uneventfully. Also, although there was a higher incidence of RSV illness among HIV-infected than HIV-uninfected women in our study, the recovery among the 5 HIV-infected women was uneventful.

Furthermore, RSV infection during pregnancy in our study was not associated with adverse pregnancy outcomes, including no difference in preterm birth, low birthweight, and stillbirths compared with either pregnant women who had influenza infection or those who did not develop either RSV or influenza infection during pregnancy.

Recent studies in animal models indicated that RSV respiratory tract infection of pregnant rats was associated with virus transmission across the placenta to the fetus (30%), which was associated with aberrant parasympathetic innervation and airway hyperreactivity after postnatal RSV reinfection [15, 16]. The clinical relevance of this murine model experiment warrants further investigation into the context of maternal RSV vaccine studies.

Furthermore, as was evident from our study and the study from Nepal, maternal postpartum RSV infection was commonly associated with concurrent RSV infection in their infants: 2 of 7 postpartum maternal episodes in Nepal and 14 of 27 (51.9%) in South Africa. Whereas it is uncertain whether the mothers infected their infants or vice versa or, alternatively, both might have been infected by another common source, prevention of such respiratory illness among the women through vaccination could provide further protection to their young infants against RSV infection by interrupting maternal–infant transmission. Furthermore, as asymptomatic RSV infections and milder illness that the mother chose not to seek medical care for would not have been investigated in our study, we likely underestimated the overall prevalence of RSV coinfections in mother–infant dyads.

## Supplementary Material

Supplementary Materials

## Supplementary Data

Supplementary materials are available at *Clinical Infectious Diseases* online. Consisting of data provided by the authors to benefit the reader, the posted materials are not copyedited and are the sole responsibility of the authors, so questions or comments should be addressed to the corresponding author.

## Notes

***Acknowledgments.*** The authors, on behalf of the Matflu team, thank all of the study participants and their probands; the staff of the Departments of Obstetrics, Neonatology, and Paediatrics at Chris Hani Baragwanath Academic Hospital, Soweto, South Africa, for their dedication to their patients, including our trial participants; and the study midwives, nurses, laboratory staff, counselors, and data capturers.

***Financial support.*** This work was supported by the Bill & Melinda Gates Foundation (grant number OPP1002747). There was also partial support from the South African Research Chairs Initiative of the Department of Science and Technology and National Research Foundation in Vaccine Preventable Diseases; and the Medical Research Council’s Respiratory and Meningeal Pathogens Research Unit.

***Potential conflicts of interest.*** All authors: No reported conflicts of interest. All authors have submitted the ICMJE Form for Disclosure of Potential Conflicts of Interest. Conflicts that the editors consider relevant to the content of the manuscript have been disclosed.
