# Supplementary material for: Burden of Respiratory Syncytial Virus Infection in South African Human Immunodeficiency Virus (HIV)-Infected and HIV-Uninfected Pregnant and Postpartum Women: A Longitudinal Cohort Study
Source: Clin Infect Dis. 2017 Dec 15;66(11):1658–65. doi: 10.1093/cid/cix1088 (PMC5961360; doi:10.1093/cid/cix1088)
Supplement: Supplementary Materials [file cix1088_suppl_supplementary_materials.docx]

**Supplementary material**

Influenza-like-illness criteria

Influenza-like-illness in the mothers was defined as: presence of fever (≥38C on oral measurements) or chills/rigors or feeling feverish in past <7 days, and one of the following for < 7 days duration: i) cough/sore throat/pharyngitis, or ii) muscle aches/joint aches/headaches, or iii) chest pain while breathing/feeling short of breath/difficulty breathing.

Criteria for investigating for influenza illness in infants were: i) documented fever (≥37.8C on axillary measurements for < 7 days duration) irrespective of presence of respiratory symptoms and signs, or ii) Fever (documented as ≥37.8C and/or mother’s perception that infant feverish/ hot) plus at least one sign/symptom of acute respiratory infection within the past 72hrs, or iii) at least two signs/symptoms of acute respiratory infection within the past 72hrs. Signs/symptoms of acute respiratory infection included: tachypnoea, difficulty breathing, coughing, wheezing, runny or congested nose, cyanosis, chest wall in-drawing, grunting on expiration, pus draining from ear.

**Supplementary Table 1.** Illness visits and testing for Respiratory Syncytial Virus among a longitudinal cohort of HIV-infected and HIV-uninfected women and their infants enrolled in a prospective study evaluating the efficacy of trivalent Influenza vaccine during pregnancy

|  | **HIV-infected cohort**  **2011** | **HIV-uninfected cohort 2011** | **HIV-uninfected cohort 2012** |
| --- | --- | --- | --- |
| **Overall Women** |  |  |  |
| Number enrolled | 194 | 1060 | 1056 |
| Overall illness visits, n | 449 | 1477 | 2113 |
| Overall illness visits sampled, n (%) | 431 (96.0%) | 1431 (96.9%) | 2015 (95.4%) |
| Overall illness visits samples available for RSV testing, n (%) | 401 (93.0%) | 1367 (95.5%) | 1783 (88.5%) |
| Overall illness visits samples positive for RSV, n (%) | 5 (1.3%) | 10 (0.7%) | 33 (1.9%) |
| Illness visits fulfilling ILI criteria sampled, n | 60 | 205 | 171 |
| ILI visits samples available for RSV testing, n (%) | 57 (95.0%) | 193 (94.2%) | 155 (90.6%) |
| ILI visits samples positive for RSV, n (%) | 0 | 1 (0.5%) | 1 (0.7%) |
| Illness visits during RSV season sampled, n | 265 | 841 | 1669 |
| Illness visits samples during RSV season available for RSV testing, n (%) | 248 (93.6%) | 798 (94.9%) | 1460 (87.5%) |
| Illness visits samples during RSV season positive for RSV, n (%) | 4 (1.6%) | 9 (1.1%) | 29 (2.0%) |
| **Women during pregnancy** | | | |
| Overall illness visits, n | 118 | 454 | 579 |
| Overall illness visits sampled, n (%) | 113 (95.8%) | 439 (96.7%) | 566 (97.8%) |
| Overall illness visits samples available for RSV testing, n (%) | 104 (92.0%) | 412 (93.9%) | 520 (9195%) |
| Overall illness visits samples positive for RSV, n (%) | 3 (2.9%) | 4 (1.0%) | 14 (2.7%) |
| Illness visits during RSV season sampled, n | 113 | 413 | 565 |
| Illness visits samples during RSV season available for RSV testing, n (%) | 104 (92.0%) | 386 (93.5%) | 519 (91.9%) |
| Illness visits samples during RSV season positive for RSV, n (%) | 3 (2.9%) | 4 (1.0%) | 14 (2.7%) |
| **Infants** | | | |
| Live-born infants, n | 188 | 1028 | 1021 |
| Overall illness visits, n | 448 | 1673 | 2247 |
| Overall illness visits sampled, n (%) | 420 (93.8%) | 1628 (97.3%) | 2112 (94.0%) |
| Overall illness visits samples available for RSV testing, n (%) | 395 (94.1%) | 1608 (98.8%) | 1847 (87.5%) |
| Overall illness visits samples positive for RSV, n (%) | 18 (4.6%) | 56 (3.5%) | 60 (3.3%) |
| Illness visits during RSV season sampled, n | 209 | 747 | 1565 |
| Illness visits samples during RSV season available for RSV testing, n (%) | 199 (95.2%) | 738 (98.8%) | 1385 (88.5%) |
| Illness visits samples during RSV season positive for RSV, n (%) | 13 (6.5%) | 51 (6.9%) | 54 (3.9%) |

RSV: Respiratory Syncytial Virus; ILI: influenza-like-illness.

**Supplementary Table 2.** Clinical signs and symptoms of Respiratory Syncytial Virus and Influenza illness in pregnant and post-partum HIV-infected women

|  | **RSV infected women**  **N=5** | **Influenza infected women**  **N=23** | **Neither RSV or influenza virus identified**  **N=166** | **p-value^1^** | **p-value^2^** |
| --- | --- | --- | --- | --- | --- |
| Mean age at enrolment (SD), years | 30.2 (6.4) | 26.7 (4.4) | 27.7 (5.1) | 0.15 | 0.29 |
| Primigravida, n (%) | 2 (40.0) | 2 (8.7) | 29 (17.5) | 0.14 | 0.22 |
| Hospitalized for LRTI within 15 days of viral detection, n (%) | 0 | 3 (8.7) | 3 (1.8)^3^ | 0.99 | 0.99 |
| Signs and symptoms, n (%)^4^ | N=5 | N=23 | N=403 |  |  |
| Cough | 4 (80.0) | 17 (73.9) | 286 (71.1) | 0.99 | 0.99 |
| Chills/rigors | 0 | 9 (40.9) | 55 (13.7) | 0.14 | 0.99 |
| Rhinorrhea | 4 (80.0) | 12 (52.2) | 260 (68.2) | 0.36 | 0.99 |
| History fever | 0 | 3 (13.0) | 13 (3.2) | 0.99 | 0.99 |
| Myalgia | 0 | 2 (8.7) | 47 (11.7) | 0.99 | 0.99 |
| Headache | 1 (20.0) | 15 (65.2) | 212 (52.6) | 0.13 | 0.20 |
| Sore throat | 3 (60.0) | 8 (34.8) | 164 (40.7) | 0.35 | 0.41 |
| Pneumonia | 0 | 3 (13.6) | 9 (2.5) | 0.99 | 0.99 |
| Bronchitis | 0 | 0 | 2 (0.6) | - | 0.99 |
| Pulmonary tuberculosis | 0 | 0 | 2 (0.6) | - | 0.99 |
| **Pregnancy outcomes, n (%)** | **RSV illness during pregnancy**  **N=3** | **Influenza illness during pregnancy**  **N=11** | **Neither RSV or influenza illness during pregnancy**  **N=169^5^** |  |  |
| Stillbirth | 0 | 0 | 0 |  |  |
| Preterm birth | 0 | 3 (27.3) | 22 (13.0) | 0.99 | 0.99 |
| Birthweight <2500gr | 1 (33.3) | 2 (18.2) | 24/167 (14.4) | 0.99 | 0.38 |

^1^p-value for the comparison RSV-associated illnesses vs. influenza-associated illnesses.

^1^p-value for the comparison RSV-associated illnesses vs. neither RSV or influenza-associated illnesses.

^3^All women who did not have an RSV or Influenza episode were included.

^4^Based on the number of samples available for testing.

^5^Women with known fetal outcomes who did not have any RSV or influenza-associated illness during pregnancy.

RSV: Respiratory Syncytial Virus; LRTI: lower respiratory tract infection; SD: standard deviation.
